# MiR-21-3p Plays a Crucial Role in Metabolism Alteration of Renal Tubular Epithelial Cells during Sepsis Associated Acute Kidney Injury via AKT/CDK2-FOXO1 Pathway

**DOI:** 10.1155/2019/2821731

**Published:** 2019-05-16

**Authors:** Zhuoyong Lin, Zhongwei Liu, Xi Wang, Chuan Qiu, Shixiang Zheng

**Affiliations:** ^1^Department of Anesthesiology, Fujian University of Traditional Chinese Medicine Affiliated People's Hospital, Fuzhou, Fujian 350001, China; ^2^Department of Cardiology, Shaanxi Provincial People's Hospital, Xi'an, Shaanxi 710068, China; ^3^Department of Obstetrics and Gynecology, The Second Xiangya Hospital, Central South University, Changsha, Hunan 410011, China; ^4^Department of Biostatistics & Bioinformatics, School of Public Health & Tropical Medicine, Tulane University, New Orleans, LA 70118, USA; ^5^Division of Critical Care Medicine, Union Hospital of Fujian Medical University, Fuzhou, Fujian 350001, China

## Abstract

**Objective:**

Sepsis and associated acute kidney injury (SAKI) are determined to be closely related to poor prognosis. Because the metabolic alterations of tubular epithelial cells (TECs) are crucial for the occurrence and development of SAKI, we carried out this present study to identify the metabolism changes of TECs during SAKI and relevant mechanisms.

**Methods:**

Rat SAKI model and rat tubular epithelial cell line were used in our study. ELISA was used to determine the serum cytokines levels. Protein expressions were examined with Western-Blotting and the transcriptions of RNAs were determined with qRT-PCR. ADP/ATP assay and Oil Red O staining were used to examine the energy and lipid metabolism, respectively. Dual-luciferase reporter assay was carried out to determine the interactions between miRNA and specific proteins. Cell cycle arrest and apoptosis were determined with flow cytometry.

**Results:**

Sepsis and AKI were induced 12 h after CLP. Energy and lipid metabolism reduced significantly while FOXO1 levels increased remarkably in TECs during SAKI. The expressions of both AKT and CDK2 and the transcriptions of relevant mRNAs reduced significantly in TECs during SAKI while miR-21-3p expression increased remarkably. Both AKT and CDK2 were determined as the direct targets of miR-21-3p. Furthermore, by in vitro experiments, it was demonstrated that FOXO1 levels were regulated by miR-21-3p in TECs via AKT/CDK2 and AKT/CDK2-FOXO1 pathway was crucial in the regulations of miR-21-3p on lipid metabolism, cell cycle arrest, and apoptosis of TECs.

**Conclusions:**

MiR-21-3p mediates metabolism and cell fate alterations of TECs via manipulating AKT/CDK2-FOXO1 pathway, and that is crucial in the regulation of energy metabolism of TECs during SAKI.

## 1. Introduction

Sepsis remains as the leading cause of mortality in Intensive Care Units and causes a huge economic burden worldwide [1, 2]. The clinical prognosis depends on the degree of organ failure and the extent to which organ function is restored. To date, there are few effective treatments for sepsis and related organs dysfunction besides instant antibiotic treatment and supportive therapy such as fluid resuscitation [3, 4]. Therefore, in order to find better prevention and treatment strategies for sepsis, it is crucial to explore the further mechanisms which cause cell dysfunction and organ failure during sepsis.

The kidney is one of the most vulnerable organs in sepsis and sepsis associated acute kidney injury (SAKI) is determined to be closely related to poor prognosis and progress to chronic kidney disease (CKD) [5–8]. Renal tubular epithelial cell (TEC) acts as a central role in the occurrence and development of SAKI [9–11]. Epithelium injury and dysfunction caused by adaptive and/or maladaptive responses were attributed as increasing importance as the mechanisms of such process. Previous study indicated that TECs suffer rarely from cell death but undergo functional shutdown during SAKI [12–14]. Although recent studies presented by Sureshbabu and colleague indicated that mitochondrial injury may promote acute kidney injury in sepsis [15], there are few studies focusing on energy metabolism of TECs during this process and associated mechanisms. For energy metabolism is crucial for cell's function, the metabolism alteration of TECs during SAKI needs to be further uncovered. Moreover, as a main nucleus factor that regulates transcriptions of genes related to cell metabolism, how FOXO1 participates in the progress of metabolic changes of TECs in such a situation as SAKI is needed to be further clarified.

Factors involved in the pathophysiology of SAKI are multiple, while microRNA plays an important part [16, 17]. MicroRNA is known to be one kind of noncoding RNAs that can regulate specific genes expression posttranscriptionally by targeting their mRNAs. Recent studies have revealed that numerous microRNAs involved in the process of sepsis and associated immune suppression, organ malfunction, and metabolism dysregulation [18–22]. These microRNAs serve as either extracellular or intracellular effector molecules targeting special mRNAs to manipulate metabolism and function of cells. Many microRNAs have been studied in either animal or human researches of specific kidney disease such as ischemia reperfusion injury and fibrosis [23–26]. However, little has been uncovered of the roles that microRNA plays in the cellular metabolism alteration of TECs in SAKI. MiR-21 is ubiquitously expressed in many organs such as heart and kidney in mammals [17, 27]. Studies indicated that miR-21 is a player in podocyte apoptosis and the progression of kidney fibrosis [28, 29]. Moreover, miR-21-3p was determined to be the key regulator at the intersection of inflammation, energy metabolism, redox biology, and cancer biology [30–33]. Therefore, it is important to find out whether and how the miR-21-3p involves in the process of SAKI, especially to unveil its role in the functional and metabolic alterations of TECs.

In our present study, the rat CLP sepsis animal model and rat renal tubular epithelial cell line were used to verify the metabolism changes of TECs during SAKI and the role miR-21-3p plays in this process. Moreover, we identified a crucial intracellular pathway that mediated miR-21-3p's effects on metabolism and cell fate determination of TECs.

## 2. Material and Methods

### 2.1. Animals

SPF Sprague-Dawley (SD) rats weighting from 200 to 300 grams supplied by Hubei Institute of Experimental Animal were used in the present study. The animal study protocols were approved by the Institutional Animal Use and Care Committee of Fujian Medical University Union Hospital. The rats were raised in a temperature and humidity control laminar flow room with the artificial 12-12 light cycle. All animals had free access to tap water and standard rat chow. The rats were divided into control group (Ctrl group), cecal ligation and puncture group (CLP group), and CLP + AS1842856 group. All the specimens were harvested when the rats were sacrificed with CO2. For the Ctrl group, the specimens were harvested 12 h after the operation. For the CLP group and the CLP + AS1842856 group, the rats were further divided into 12 h group, 18 h group, and 24 h group of which the specimens were collected on 12th hour, 18th hour, and 24th hour, respectively, after the surgery. Meanwhile, 12 h, 18 h, and 24 h marked on the horizontal axis were used to represent the 12 h group, 18 h group, and 24 h group, respectively, in the figures.

### 2.2. Surgery Protocols

The CLP procedure was carried out as previously described. Briefly, the rats were anesthetized with isoflurane and the cecum was isolated. For the CLP group, the ligation was at the position of 50% of the cecum and the cecum was punctured in a through-and-through way at the midpoint of between the ligation and the end tip of the cecum. A small amount of feces was excluded before closing the abdominal cavity and prewarmed normal saline (37°C, 5 ml/100g) was injected subcutaneously for resuscitation. For the CLP + AS1842856 group, CLP was performed and the AS1842856 (Sigma) contained saline (0.2mg/ml, 37°C, 5 ml/100g) was administered for resuscitation. For the Ctrl group, the cecum was located after being anesthetized without ligation and puncture and the rest of the steps were the same as the CLP group.

### 2.3. TEC Isolation

TECs were isolated as described before. Briefly, the cortex of the kidney was ground on 80- and 100-mesh screen and the residue on the 100-mesh screen was digested with 0.2% trypsin (Hyclone). After being washed with PBS for 3 times, the TECs were sorted with magnetic cell sorting technique with FITC anti-cytokeratin 18 antibody (Abcam) and anti-FITC microbeads (Miltenyi Biotec).

### 2.4. Cell Treatment

Both rat TECs (NRK52E) and HEK 293 cell lines were cultured in DMEM (Hyclone) supplemented with 10%FBS+1% (Penicillin-Streptomycin Solution). All the cells were cultured at 37°C with 5% CO2. The miR-21-3p mimic, miR-21-3p inhibitor, their negative control (GenePharma) oligonucleotides, and vectors were transfected to the cells cultured to 70-80% confluence using Lipofectamine™2000 according to the manufacturer's instructions (Invitrogen). The AKT overexpression plasmid (AKT OEX P, GenePharma) and CDK2 overexpression plasmid (CDK2 OEX P, GenePharma) were also cotransfected to the TECs with miR-21-3p mimic accordingly using Lipofectamine™2000. For the FOXO1, AKT, and CDK2 inhibitors treated groups, the cells were incubated with one of AS1842856 (1uM, MCE), GSK2141795 (2.5uM, MCE), and CVT-313 (1uM, MCE) along with the serum free DMEM accordingly for 12 h after the transfection before sampling. For the other groups, the cells were incubated with the serum free DMEM for 12 h after the transfection before sampling.

### 2.5. ELISA, White Blood Cell Count, and Biochemistry Assay

White blood cell count (WBC) was carried out with BC-2800 Fully Automatic Hematology Analyzer (Mindray). IL-6 and TNF-a were measured with commercial ELISA kits according to the manufacturer's instructions (Elabscience). Biochemistry assays were carried out with 7020 Automatic Biochemical Analyzer (HITACHI).

### 2.6. Western Blotting Assay

Western Blotting assay was used to examine the protein expression as previously described. For the cell derived samples, RIPA buffer containing a protease inhibitor (Sigma) was used to extract the total protein. For nuclei derived samples, the proteins were extracted and prepared using CelLytic™ NuCLEAR™ Extraction Kit (Sigma) in accordance with the manufacturer's instructions. Protein lysates were separated with SDS-PAGE and transferred to the PVDF membranes (Millipore). After that, the membranes were cut into pieces according to the molecular weights of target proteins and incubated with the primary antibodies [AKT rabbit polyclonal antibody, 1:1000 (ProteinTech); CDK2 rabbit polyclonal antibody, 1:800 (ProteinTech); FOXO1 rabbit polyclonal antibody, 1:1000 (ProteinTech); phosphate FOXO1 rabbit polyclonal antibody, 1:800 (Abcam)] at 4°C for 12 h. The AlexaFluor 680/790-labeled goat anti-rabbit IgG antibody (1:10000, LI-COR Biosciences) was used as the secondary antibody. The blots were visualized by LI-COR Odyssey Infrared Imaging System (LI-COR Biosciences).

### 2.7. Real-Time qPCR

mRNA quantitation was carried out using real-time qPCR. In short, total RNA from isolated TECs and in vitro cultured cells were collected and purified according to the manufacturer's instructions (Invitrogen). cDNA was synthesized with First Strand cDNA Synthesis Kit (TOYOBO). A StepOne Real-Time PCR System (Invitrogen) was used to perform the relative real-time qPCR with Thunderbird SYBR qPCR Mix (TOYOBO). The GAPDH and U6 were used as the internal control for mRNA and miRNA, respectively, as described previously. The gene expression levels were determined by the ΔΔCt method. The specific primers of the target mRNAs and internal controls were the following: Rat AKT, 5′- GCTCTTCTTCCACCTGTCTCG -3′ and 5′- CACAGCCCGAAGTCCGTTA -3′; Rat CDK2, 5′- TGACGGGAGAAGTTGTGG -3′ and 5′- CGATGTTAGGGTGATTGAG-3′; Rat FOXO1, 5′- CCATGCCTCACACATCTGCC -3′ and 5′- TTAAAATCCAAGGTATCTCCGTCCA -3′; Rat GAPDH, 5′- ACAGCAACAGGGTGGTGGAC -3′ and 5′- TTTGAGGGTGCAGCGAACTT -3′; Rno-miR-21-3p, 5′- TGCGCCAACAGCAGTCGATGGG -3′ and loop primer 5′-GTCGTATCCAGTGCAGGGTCCGAGGTATTCGCACTGGATACGAC GACAGCCC -3′; U6, 5′- CGCTTCGGCAGCACATATAC -3′ and 5′- AAATATGGAACGCTTCACGA -3′.

### 2.8. Oil Red O Staining

Both the frozen tissue sections and cell slides were fixed with 4% paraformaldehyde for 15 min. All the samples were stained with Oil Red O solution in 60% isopropanol for 10 min and washed 3 times with distilled water. Mayer hematoxylin was used for counterstaining and the stained samples were visualized and photographed by light microscopy.

### 2.9. ADP/ATP Ratio Assay

The ADP/ATP ratio assay of isolated TECs was carried out using ADP/ATP Ratio Assay Kit according to manufacturer's instruction (Sigma). A luminometer was used to read the luminescence for ATP (relative light units A, RLUA), background (RLUB), and ADP (RLUC). ADP/ATP Ratio = (RLUC-RLUB)/RLUA.

### 2.10. Dual-Luciferase Reporter Assay

HEK 293 cells were seeded in 24-well plates at approximately 10^5^ per well and cultured overnight. The wildtype or mutant AKT promoter-luciferase plasmids (0.1 *μ*g per well of pYr-rat-AKT-3UTR or pYr-rat-AKT-3UTR-mut plasmids) and wildtype or mutant CDK2 promoter-luciferase plasmids (0.1 *μ*g per well of pYr-rat-CDK2-3UTR or pYr-rat-CDK2-3UTR-mut plasmids) were transfected to different groups of cells mediated by Lipofectamine 2000 (Invitrogen). Cells were also cotransfected with either 0.4 *μ*g miR-21-3p mimics or 0.4 *μ*g miR-21-3p NC accordingly (Genepharma). Cotransfection with 0.02*μ*g of Renilla luciferase reporter was used to standardize the transfection efficiency (Genechem). Luciferase activities were quantified using a dual-luciferase reporter assay system (Promega). All the experiments were executed in triplicate.

### 2.11. Flow Cytometric Analysis

An AnnexinV-APC/7-AAD double staining kit (Keygen) was used to determine the apoptosis rate of TECs according to manufacturer's instructions. The cell cycle of TECs was verified with the Cell Cycle kit (Multiscience Biotech) by cytometry. The cytometry was executed with Beckman CytoFLEX FCM.

### 2.12. Statistical Analysis

Quantitative data were presented as the mean±SDs. GraphPad Prism 6 was used to execute the statistical analysis. Statistically significant differences in the mean values were assessed using Student's t-test or one-way analysis of variance with Tukey's multiple comparisons test for multiple comparisons. A* P* value <0.05 was considered statistically significant.

## 3. Results

**Figure 1:**
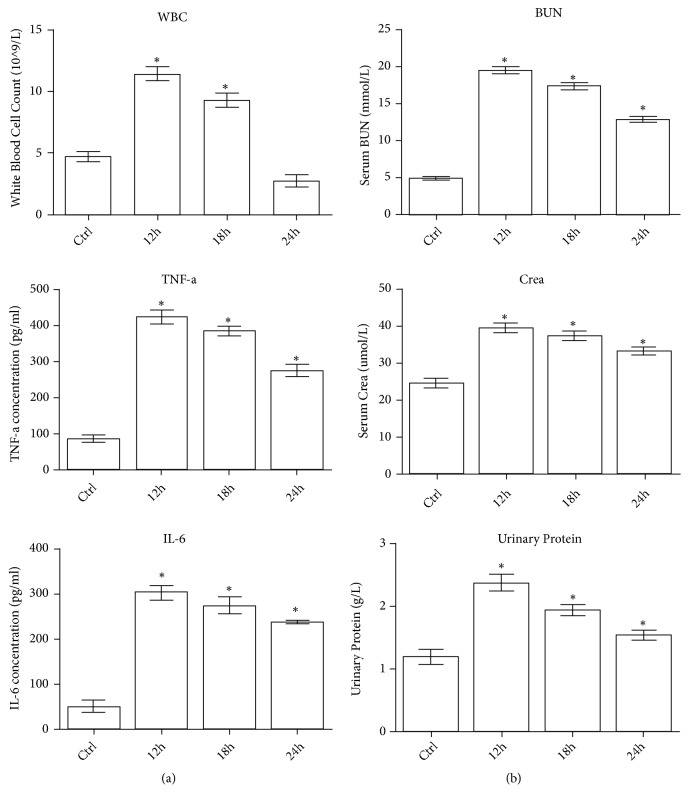
Representative parameters for sepsis and AKI in different rat groups. (a) Representative parameters for sepsis in Ctrl and CLP groups (WBC: White Blood Cell Count; *∗*: P<0.05 vs. Control). (b) Representative parameters for AKI in Ctrl and CLP groups (BUN: blood urea nitrogen, Crea: blood creatinine, and *∗*: P<0.05 vs. Control).

*(1) Sepsis and AKI Were Induced 12 h after CLP*. It is important to learn whether sepsis and associated AKI (SAKI) were induced in CLP rat model and when that will happen. As we set up the CLP rat model, we examined several biomarkers to find out whether and when sepsis and SAKI would be induced after the surgery. As it was shown in Figure 1, the white blood cell count, serum TNF-a level, and IL-6 level were increased significantly from the baseline in CLP rats 12 h after surgery (Figure 1(a)). Moreover, in CLP rats, the blood urea nitrogen level, serum creatinine level, and urine protein level also had significant risings 12 h after CLP (Figure 1(b)). For CLP is a classical way to establish sepsis animal model, it can be demonstrated from the results that sepsis and SAKI were induced in CLP rats 12 h after CLP and the animal model can be used for further study.

**Figure 2:**
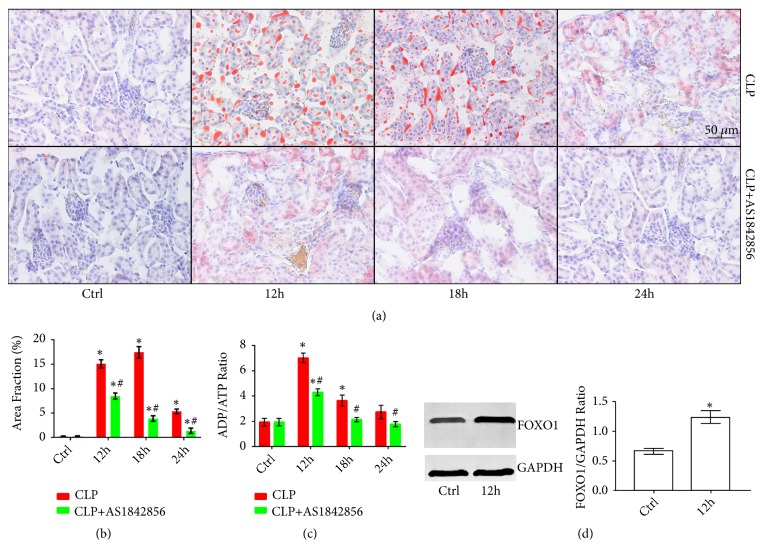
Alterations of metabolism and FOXO1 expression in TECs during SAKI. (a) Representative images of Oil Red O staining of kidney sections from different rat groups at each time point. (b) Quantitative analysis of Oil Red O staining (*∗*: P<0.05 vs. Control; #: P<0.05 vs. CLP group at the same time point). (c) Quantitative analysis of ADT/ATP Ratio of TECs from different rat groups at each time point (*∗*: P<0.05 vs. Control; #: P<0.05 vs. CLP group at the same time point). (d) Representative Western-Blotting results of FOXO1 of TECs from Ctrl and CLP groups at 12 h after the surgery (*∗*: P<0.05 vs. Control).

*(2) Metabolic Activity and FOXO1 Expression Were Altered in TECs during SAKI*. Oil Red O staining and ADP/ATP Ratio Assay were used to further investigate the alterations of metabolic activity of TECs during SAKI. Firstly, we carried out Oil Red O staining on kidney specimens of the rats in both CLP and CLP+AS1842856 groups at different time points to find out the changes of TECs lipid metabolism. By measuring Oil Red O stained lipid droplet area, it can be demonstrated from the results that lipid droplets increased significantly 12 h after the surgery and the peak appeared at 18 h after the surgery in the CLP group. Moreover, lipid droplets of the CLP+AS1842856 group also increased significantly after the CLP procedure but the area fractions were remarkably lower than that of the CLP group at each time point (Figures 2(a) and 2(b)). Furthermore, ADP/ATP Ratio Assay was carried out to investigate the metabolic activity of isolated TECs in both CLP and CLP+AS1842856 groups. As shown in Figure 2(c), ADP/ATP ratios increased significantly at both 12 h and 18 h after CLP in CLP group while that only significantly increased at 12 h after CLP in TECs treated with the FOXO1 inhibitor AS1842856. Moreover, ADP/ATP ratios of TECs in CLP+AS1842856 group were remarkably lower compared to that in CLP group at each time point (Figure 2(c)). FOXO1 levels in isolated TECs were further examined with West-Blotting technique. It could be found out that FOXO1 level increased remarkably 12 h after CLP (Figure 2(d)). All the results above demonstrated that the energy metabolic activity of TECs was suppressed as intracellular FOXO1 level rose during SAKI and the suppression of metabolic activity of TECs can be reversed as the FOXO1 was inhibited.

**Figure 3:**
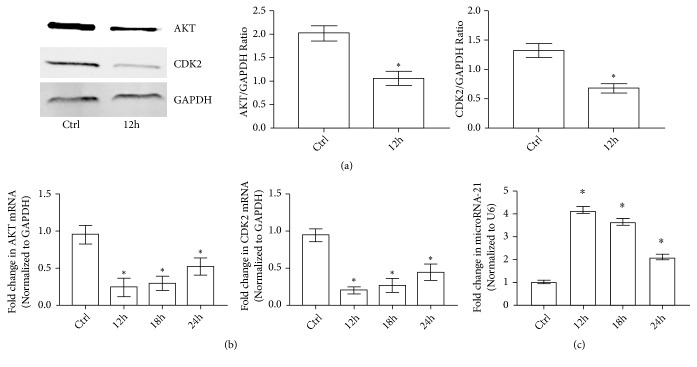
The transcriptions and expressions of AKT and CDK2 in TECs during SAKI. (a) Representative Western-Blotting results of AKT and CDK2 of TECs from Ctrl and CLP groups at 12 h after the surgery (*∗*: P<0.05 vs. Control). (b) Quantitative analysis of qRT-PCR results of AKT mRNA, CDK2 mRNA, and miR-21-3p transcription levels in TECs from different rat groups at each time point (*∗*: P<0.05 vs. Control).

*(3) The Expressions of AKT and CDK2 and MiR-21-3p Level in TECs Were Altered during SAKI*. It has been well uncovered that inhibition of AKT and CDK2 can effectively enhance the intracellular FOXO1 accumulation. Here we hypothesized that AKT and CDK2 were downregulated in TECs during SAKI and the hypothesis was confirmed by Western-Blotting as both of the expressions of AKT and CDK2 were decreased significantly in TECs 12 h after CLP (Figure 3(a)). Moreover, with qRT-PCR, it can be demonstrated that both the AKT and CDK2 mRNA levels in isolated TECs were decreased significantly 12 h after the surgery (Figure 3(b)). The results mentioned above indicated that the expressions of both AKT and CDK2 were inhibited during SAKI. To further find out how the expressions of AKT and CDK2 were regulated, two target prediction programs (TargetScan and miRanda) were used to investigate the potential miRNA and miR-21-3p was predicted to interact with both the 3′UTR of both the AKT and CDK2 mRNA. Next, with qRT-PCR, it was found out that the miR-21-3p level increased significantly 12 h after CLP (Figure 3(c)). It was suggested from the results that miR-21-3p may act a crucial role in altering the intracellular AKT and CDK2 levels in TECs during SAKI.

**Figure 4:**
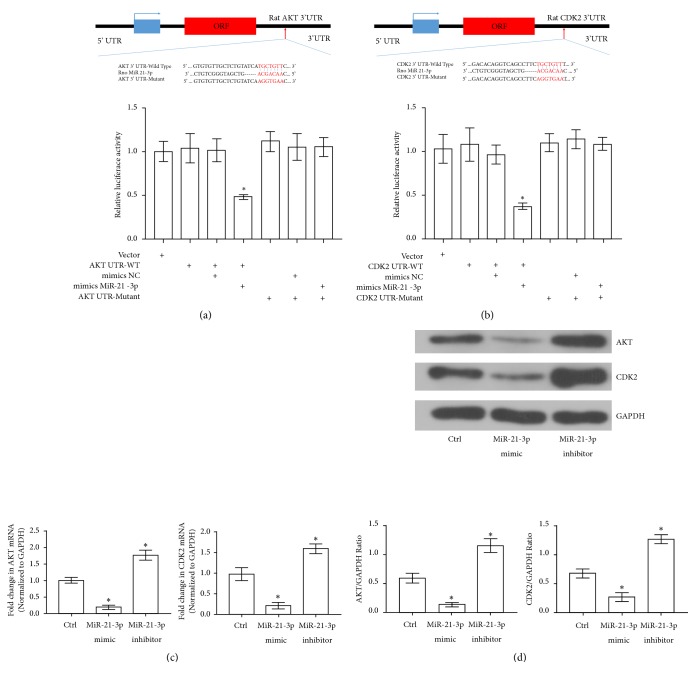
Effects of miR-21-3p on transcriptions and expressions of AKT and CDK2. (a) Representative results of luciferase reporter assay for AKT and miR-21-3p (*∗*: P<0.05 vs. Vector). Putative miR-21-3p binding sequence in the 3′-UTR of AKT mRNA. The mutation was generated in the AKT 3′-UTR sequence in the complementary site of the miR-21-3p seed region. (b) Representative results of luciferase reporter assay for CDK2 and miR-21-3p (*∗*: P<0.05 vs. Vector). Putative miR-21-3p binding sequence in the 3′-UTR of CDK2 mRNA. The mutation was generated in the CDK2 3′-UTR sequence in the complementary site of the miR-21-3p seed region. (c) Quantitative analysis of qRT-PCR results of AKT mRNA and CDK2 mRNA transcription levels in NRK52E cells from different groups (*∗*: P<0.05 vs. Control). (d) Representative Western-Blotting results of AKT and CDK2 expression levels in NRK52E cells from different groups (*∗*: P<0.05 vs. Control).

*(4) MiR-21-3p Directly Targeted AKT and CDK2 in TECs*. For AKT and CDK2 levels were negatively related with miR-21-3p in TECs during SAKI, it was important to find out whether AKT and CDK2 were the direct targets of miR-21-3p. A dual-luciferase reporter assay was carried out to determine the relationships between miR-21-3p and both AKT and CDK2. Wild-type (WT) or mutant (MUT) AKT-3′ UTR and CDK2-3′ UTR were inserted into luciferase reporter vectors. Our results have shown that miR-21-3p led to decreased luciferase activity of both the WT-AKT-3′ UTR and WT-CDK2-3′ UTR, whereas the luciferase activities of the MUT-AKT-3′ UTR and MUT-CDK2-3′ UTR were almost unchanged (Figures 4(a) and 4(b)). Moreover, with qRT-PCR and Western-Blotting assay, our results demonstrated that upregulation of miR-21-3p led to decrease in both AKT and CDK2 transcription and protein levels in TECs, whereas downregulation of miR-21-3p resulted in the opposite way (Figures 4(c) and 4(d)). It can be determined from the results above that miR-21-3p directly downregulated both AKT and CDK2 in TECs.

**Figure 5:**
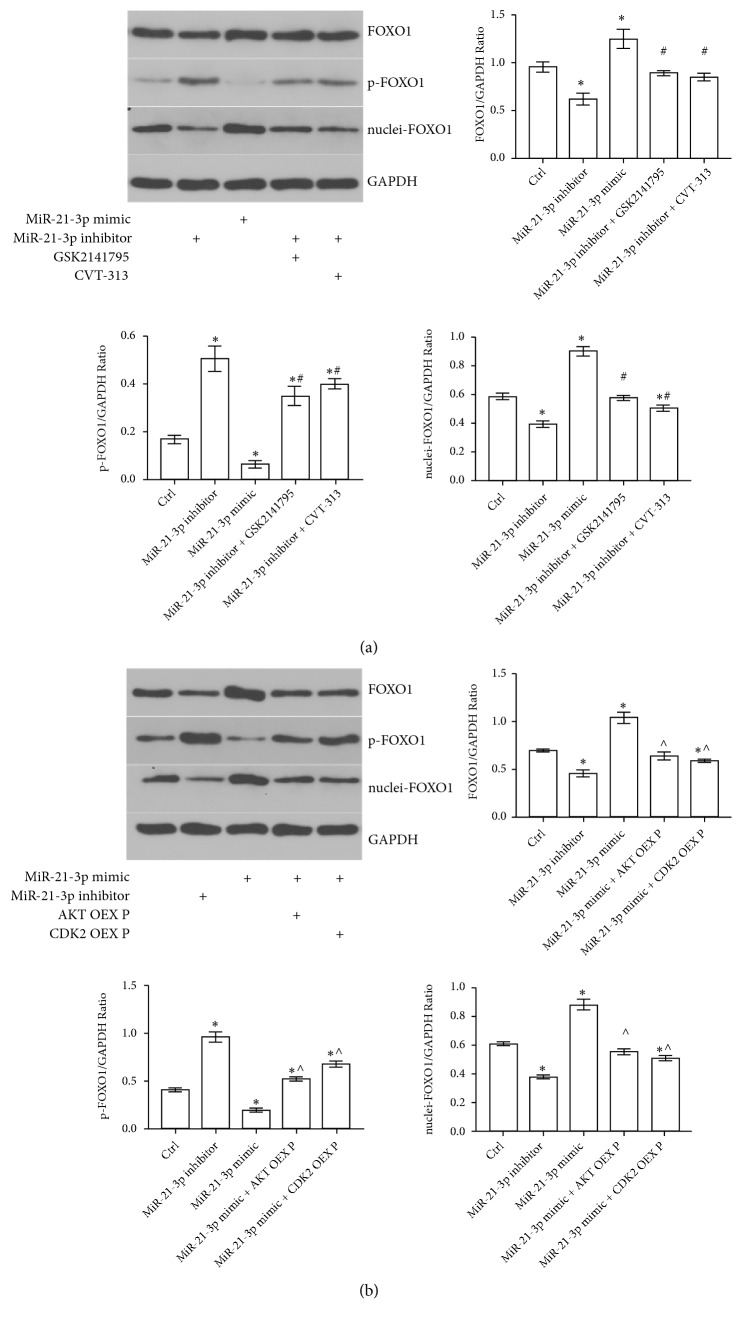
Effects of miR-21-3p expression and either AKT or CDK2 activity on FOXO1 level in TECs. (a, b) Representative Western-Blotting results of FOXO1, phosphate FOXO1 (p-FOXO1), and intranuclei FOXO1 (nuclei-FOXO1) expression levels in NRK52E cells with diverse treatments (AKT OEX P: AKT overexpression plasmid; CDK2 OEX P: CDK2 overexpression plasmid; *∗*: P<0.05 vs. Control; #: P<0.05 vs. miR-21-3p inhibitor group; ^∧^: P<0.05 vs. miR-21-3p mimic group).

*(5) FOXO1 Levels Were Regulated by miR-21-3p in TECs via AKT/CDK2*. Because AKT and CDK2 are both intracellular molecules that promote phosphorylation and they can be regulated by miR-21-3p, we carried out Western-Blotting to determine whether intracellular FOXO1 levels could be altered by miR-21-3p in TECs. It can be demonstrated from our results that, when transfected with miR-21-3p inhibitor, total intracellular FOXO1 level and intranuclei FOXO1 (nuclei-FOXO1) level decreased remarkably whilst phosphorylated FOXO1 (p-FOXO1) increased significantly in TECs. Moreover, transfection with miR-21-3p mimic resulted in the opposite way. Additionally, either AKT inhibitor GSK2142795 or CDK2 inhibitor CVT-313 could reverse the effects of miR-21-3p inhibitor on FOXO1 levels in TECs (Figure 5(a)). Furthermore, transfection of either AKT overexpression plasmid or CDK2 overexpression plasmid to TECs reversed the increase of intracellular FOXO1 level induced by miR-21-3p mimic (Figure 5(b)). Overall, our results suggested that miR-21-3p regulated FOXO1 levels via both AKT and CDK2 in TECs.

**Figure 6:**
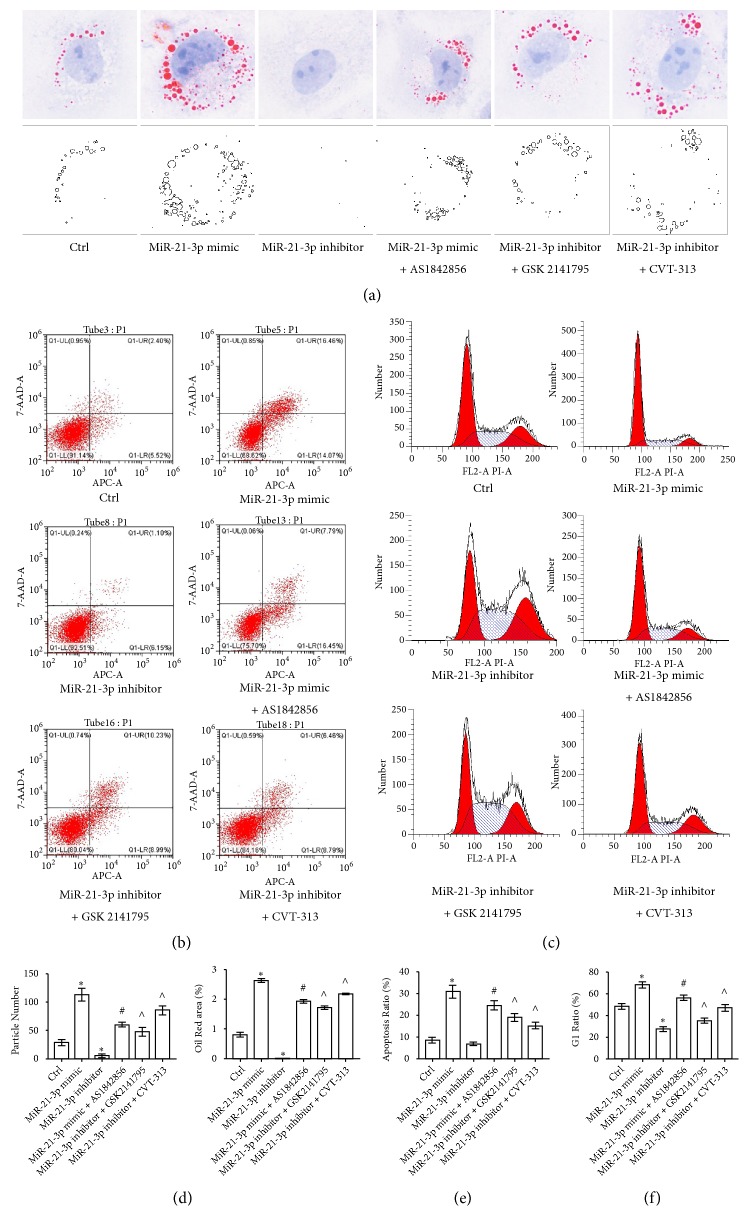
Effects of miR-21-3p and relative AKT/CDK2-FOXO1 pathway on lipid metabolism, cell cycle arrest, and apoptosis of TECs. (a) Representative images of Oil Red O staining of NRK52E cells with different treatments. (b) Representative results of flow cytometry for apoptosis of NRK52E cells with different treatments. (c) Representative results of flow cytometry for cell cycle arrest of NRK52E cells with different treatments. (d) Quantitative analysis of Oil Red O staining (*∗*: P<0.05 vs. Control; #: P<0.05 vs. miR-21-3p mimic group; ^∧^: P<0.05 vs. miR-21-3p inhibitor group). (e) Quantitative analysis of flow cytometry for apoptosis (*∗*: P<0.05 vs. Control; #: P<0.05 vs. miR-21-3p mimic group; ^∧^: P<0.05 vs. miR-21-3p inhibitor group). (f) Quantitative analysis of flow cytometry for cell cycle arrest (*∗*: P<0.05 vs. Control; #: P<0.05 vs. miR-21-3p mimic group; ^∧^: P<0.05 vs. miR-21-3p inhibitor group).

*(6) AKT/CDK2-FOXO1 Pathway Was Crucial for the Regulations of miR-21-3p on Lipid Metabolism, Cell Cycle Arrest, and Apoptosis of TECs*. It has been suggested from previous study that FOXO1 could regulate lipid metabolism, cell cycle arrest, and apoptosis of eukaryotic cells as an important nuclear factor. For our present results had demonstrated that miR-21-3p regulated FOXO1 levels via both AKT and CDK2 in TECs, we carried out Oil Red O staining and flow cytometry to determine whether and how miR-21-3p could regulate the cell biology indicators mentioned above. By measuring Oil Red O stained lipid droplet number and area, it can be demonstrated from the results that lipid droplet increased significantly when TECs were transfected with miR-21-3p mimic and FOXO1 inhibitor AS1842856 reduced that effect of miR-21-3p mimic on TECs. Meanwhile, lipid droplet decreased remarkably in TECs transfected with miR-21-3p inhibitor and either AKT inhibitor GSK2142795 or CDK2 inhibitor CVT-313 could reverse that phenomenon (Figures 6(a) and 6(d)). Moreover, with flow cytometry, it could be determined that transfection of miR-21-3p mimic could lead to significant increasing in apoptosis rate and cell cycle G1 ratio of TECs which also could be reduced when the transfected TECs were treated with AS1842856. Furthermore, transfection of miR-21-3p inhibitor resulted in remarkable decreasing of apoptosis rate and cell cycle G1 ratio of TECs whilst these effects could be reversed by treating the TECs with either GSK2142795 or CVT-313 (Figures 6(b), 6(c), 6(e), and 6(f)). After all, it can be demonstrated from the results that miR-21-3p could regulate lipid metabolism, cell cycle arrest, and apoptosis of TECs and AKT/CDK2-FOXO1 was a crucial pathway that mediated that process.

## 4. Discussion

Sepsis is a leading cause of organ failure and death in ICU worldwide, whilst SAKI is one of the major independent factors influencing the mortality and morbidity of critically ill patients. Efforts have been made to unveil the exact mechanisms in order to find out effective treatments for SAKI. However, to date, little progress has been achieved and the mechanisms inducing AKI during sepsis are still mysterious. In our previous study that aimed to look for the possible mechanisms of SAKI, we found out that the alteration of functional status and structure of TECs was critically related to the occurrence of SAKI [34, 35]. These findings are consistent with the conclusions of previous studies focusing on AKI. Recent studies also revealed that energy substrate metabolism changed in many organs during sepsis whilst biological reactions decreased significantly in kinds of cells [36, 37]. Because TEC acts as one crucial role in the development of SAKI, it is important to uncover the metabolic alteration of TECs during this process. For energy substrate metabolism changed from glucose to predominantly lipid metabolism in sepsis [36], our present study was carried out to unveil the lipid metabolism of TECs during SAKI and associated mechanisms. It can be revealed from our results that lipid metabolism remarkably reduced as lipid accumulated and ADP/ATP ratio significantly increased in TECs of SAKI rat model induced by CLP.

FOXO1 is an intracellular super transcription factor which modulates numerous cell activities, such as metabolism, apoptosis, and cell circle arrest and maintains tissue homeostasis and changes in response to various stimulations [38, 39]. Recent study revealed that FOXO1 positively regulated intracellular lipid droplet formation and growth [40]. Here in our present study, it can be found out that FOXO1 level rose remarkably as lipid droplet accumulated in TECs of SAKI rat model. Moreover, FOXO1 specific inhibitor AS1842856 improved the energy metabolism of TECs of SAKI rats. Taken together, our results suggest that FOXO1 functions as an important regulatory molecule that mediates substances and energy metabolism alteration in TECs during SAKI.

The activity of FOXO1 is regulated by sophisticate mechanisms which have not been determined thoroughly yet including its abundance, posttranscriptional modifications, and subcellular localization. Previous studies provide evidence that phosphorylation is an important posttranscriptional modifier which induces the shuttling of FOXO1 to cytoplasm and mediates the ubiquitination and degradation of FOXO1. AKT has been well established to be one of the most important manipulative factors of FOXO1 which induce its phosphorylation and degradation [41]. Moreover, recent study has indicated that CDK2 also can bind to and phosphorylate FOXO1 at Ser^256^ residue [42]. Therefore, we hypothesized that the increase of FOXO1 in TECs during SAKI was due to the decrease of intracellular AKT and CDK2. With Western-Blotting and qRT-PCR, it can be determined from the results that both the transcription and expression of AKT and CDK2 in TECs isolated from SAKI rats decreased remarkably. Thus, our present results confirm our hypothesis.

Studies have determined that numerous signal pathways could enhance the activity of intracellular proteins that mediate phosphorylation such as AKT and CDK2. However, there are not so many studies that focus on how the downregulation of intracellular phosphorylated proteins occurs, especially in TECs. MicroRNAs are known as small noncoding RNAs which can destabilize target mRNAs distinctively and attenuate the transcription and expression of the target proteins. Recent study indicates that miR-21 promotes lipid and glucose metabolic disorders in hepatocytes [43]. In our present study, our findings indicated that the transcription level of miR-21-3p increased significantly in TECs of SAKI group. Moreover, it can be determined from the results that miR-21-3p directly targets both AKT and CDK2 and regulates their expressions in TECs. Previous study that was carried out by Dellago and colleagues revealed that transfection of normal human cells with miR-21 resulted in lower angiogenesis and less cell proliferation mirrored by upregulation of p21^CIP1^ and downregulation of CDK2 [44]. In addition, numerous studies have well determined that miR-21 regulates intracellular AKT amount and activity. The results of this present study are consistent with previous studies and further uncover the associated mechanisms.

From all the results above, it can be concluded that miR-21-3p alters the metabolic process of TECs by upregulating FOXO1 via directly targeting AKT and CDK2 during SAKI. To further verify this conclusion, in vitro studies using rat renal tubular epithelial cell line (NRK52E) were carried out. It can be demonstrated from the results that miR-21-3p could enhance both the total FOXO1 expression and nuclei-FOXO1 level via targeting AKT and CDK2 in TECs. Moreover, miR-21-3p upregulated lipid droplet accumulation of TECs via AKT/CDK2-FOXO1 pathway. The results of in vitro studies further confirmed our conclusion. Previous researches demonstrated that increasing FOXO1 level could lead to cell cycle G1 arrest in kinds of tumor cells [45, 46]. Liu and colleagues also revealed that overexpression of FOxO1 caused cell cycle G1 arrest of mesangial cells of rat kidney [47]. Moreover, studies also indicated that FOXO1 enhanced cell apoptosis while tight regulation of FOXO1 was essential for the maintenance of various tumor cells [48, 49]. Here in our present in vitro research, we examined the effects of miR-21-3p and associated AKT/CKD2-FOXO1 pathway on apoptosis and cell cycle arrest of TECs additionally. Our results were consistent with previous studies that miR-21-3p induced cell cycle G1 arrest and apoptosis of TECs via AKT/CDK2-FOXO1 pathway.

Taken together, it can be revealed from our present study that miR-21-3p could manipulate cell metabolic process and cell fate regulated by FOXO1 of TECs via directly targeting AKT and CDK2, and this mechanism plays an important regulatory role in the energy metabolism of TECs in SAKI. Nevertheless, there are some limitations in our research such as the following: (1) owing to the fact that one microRNA can have multiple targets, further researches should be conducted to verify whether there were some other pathways through which miR-21-3p could regulate the energy metabolism of TECs. (2) Although our results revealed the relationship between miR-21-3p and the alteration of energy metabolism of TECs in SAKI and associated mechanism, it is needed to verify whether this effect is protective or harmful to the long-term prognosis of SAKI. (3) The specific mechanisms that induced the upregulation of miR-21-3p in TECs during SAKI are needed to be further investigated.

In summary, our findings are the first to reveal that miR-21-3p mediates metabolism and cell fate alteration of TECs via manipulating AKT/CDK2-FOXO1 pathway, and this mechanism plays a novel role in the regulation of energy metabolism of TECs during SAKI. These findings may help to illuminate a better understanding of the exact mechanisms of SAKI and provide a basis for new strategies for further effective treatment of that disease.

## Data Availability

The data used to support the findings of this study are either included within the article or available from the corresponding author upon request.
